# Development and validation of radiologic scores for guiding individualized induction chemotherapy in T3N1M0 nasopharyngeal carcinoma

**DOI:** 10.1007/s00330-021-08460-1

**Published:** 2022-01-06

**Authors:** Shan-Shan Yang, Yi-Shan Wu, Ya-Jun Pang, Su-Ming Xiao, Bao-Yu Zhang, Zhi-Qiao Liu, En-Ni Chen, Xu Zhang, Pu-Yun OuYang, Fang-Yun Xie

**Affiliations:** 1 Department of Radiation Oncology, Sun Yat-Sen University Cancer Center, State Key Laboratory of Oncology in South China, Collaborative Innovation Center for Cancer Medicine, Guangdong Key Laboratory of Nasopharyngeal Carcinoma Diagnosis and Therapy, No. 651 Dongfeng Road East, Guangzhou, 510060 China; 2 Department of Nasopharyngeal Carcinoma, Sun Yat-Sen University Cancer Center, State Key Laboratory of Oncology in South China, Collaborative Innovation Center for Cancer Medicine, Guangdong Key Laboratory of Nasopharyngeal Carcinoma Diagnosis and Therapy, No. 651 Dongfeng Road East, Guangzhou, 510060 China; 3 Cancer Center, Affiliated Hospital of Guangdong Medical University, Renmin Avenue, Xiashan District, Zhanjiang, China; 4 Department of Nuclear Medicine, Sun Yat-Sen University Cancer Center, State Key Laboratory of Oncology in South China, Collaborative Innovation Center for Cancer Medicine, Guangdong Key Laboratory of Nasopharyngeal Carcinoma Diagnosis and Therapy, No. 651 Dongfeng Road East, Guangzhou, 510060 China

**Keywords:** Nasopharyngeal carcinoma, [^18^F]FDG PET/CT, MRI, Induction chemotherapy

## Abstract

**Objectives:**

We aimed to develop and validate radiologic scores from [^18^F]FDG PET/CT and MRI to guide individualized induction chemotherapy (IC) for patients with T3N1M0 nasopharyngeal carcinoma (NPC).

**Methods:**

A total of 542 T3N1M0 patients who underwent pretreatment [^18^F]FDG PET/CT and MRI were enrolled in the training cohort. A total of 174 patients underwent biopsy of one or more cervical lymph nodes. Failure-free survival (FFS) was the primary endpoint. The radiologic score, which was calculated according to the number of risk factors from the multivariate model, was used for risk stratification. The survival difference of patients undergoing concurrent chemoradiotherapy (CCRT) with or without IC was then compared in risk-stratified subgroups. Another cohort from our prospective clinical trial (*N* = 353, NCT03003182) was applied for validation.

**Results:**

The sensitivity of [^18^F]FDG PET/CT was better than that of MRI (97.7% vs. 87.1%, *p* < 0.001) for diagnosing histologically proven metastatic cervical lymph nodes. Radiologic lymph node characteristics were independent risk factors for FFS (all *p* < 0.05). High-risk patients (*n* = 329) stratified by radiologic score benefited from IC (5-year FFS: IC + CCRT 83.5% vs. CCRT 70.5%; *p* = 0.0044), while low-risk patients (*n* = 213) did not. These results were verified again in the validation cohort.

**Conclusions:**

T3N1M0 patients were accurately staged by both [^18^F]FDG PET/CT and MRI. The radiologic score can correctly identify high-risk patients who can gain additional survival benefit from IC and it can be used to guide individualized treatment of T3N1M0 NPC.

**Key Points:**

*• [* ^*18*^ *F]FDG PET/CT was more accurate than MRI in diagnosing histologically proven cervical lymph nodes.*

*• Radiologic lymph node characteristics were reliable independent risk factors for FFS in T3N1M0 nasopharyngeal carcinoma patients.*

*• High-risk patients identified by the radiologic score based on [* ^*18*^ *F]FDG PET/CT and MRI could benefit from the addition of induction chemotherapy.*

**Supplementary Information:**

The online version contains supplementary material available at 10.1007/s00330-021-08460-1.

## Introduction

In 2020, 133,354 new cases of nasopharyngeal carcinoma were reported, accounting for 0.7% of all cancers in the world, but over 70% of patients were from Asia, with an age standardized rate (world) of 3.0 per 100,000 in China [1, 2]. Unfortunately, over 75% of patients are diagnosed with locoregionally advanced disease at presentation [3]. Despite advances in techniques, nearly 30% of patients experience treatment failure, especially distant metastasis [4]. Phase III randomized controlled trials have proven that induction chemotherapy added to concurrent chemoradiotherapy can significantly decrease the risk of distant metastasis and improve the survival of patients with locoregionally advanced nasopharyngeal carcinoma [5–7]. This treatment mode is thus the category 2A recommendation for these patients by National Comprehensive Cancer Network (NCCN) guidelines [8]. However, notably, these randomized trials did not enroll any patients staged with T3-4N0M0 or T3N1M0 at all. A retrospective study reported that patients with T3N0-1 do not benefit from induction chemotherapy [9], while male T3N1 patients with Epstein-Barr virus (EBV) DNA higher than 2000 copies/mL were the only target population for induction followed by concurrent chemoradiotherapy, as suggested by another study [10]. Therefore, the treatment modality of T3N1 nasopharyngeal carcinoma is still controversial. Although EBV DNA has been reported to have prognostic value, its extensive application is difficult in real-world practice due to the lack of recognized cutoff values and unified test standards. 2-Deoxy-2-[^18^F]fluoro-d-glucose ([^18^F]FDG) positron emission tomography/computed tomography (PET/CT) and magnetic resonance imaging (MRI) have been widely applied for the diagnosis and staging of nasopharyngeal carcinoma [11]. Given the widespread use of [^18^F]FDG PET/CT and MRI, the radiologic characteristics of the primary tumor and metastatic lymph nodes may prove useful for selecting individualized treatment.

The maximal standardized uptake value (SUVmax) of [^18^F]FDG PET/CT, related to metabolic activity, has prognostic implications and is used for risk stratification [12, 13]. The SUVmax of lymph nodes (SUVmax-N) and the lymph node-to-primary tumor SUVmax ratio are potential prognostic factors in nasopharyngeal carcinoma patients [12, 14]. However, the prognostic value of the SUVmax of the primary tumor (SUVmax-T) is in dispute [15]. Previous studies showed that ungraded radiologic extranodal extension determined by MRI had no prognostic significance in nasopharyngeal carcinoma [16, 17]. After grading the radiologic extranodal extension, the sensitivity of diagnosing pathologic extranodal extension improved in head and neck cancer [18], and the consistency of determining radiologic extranodal extension also increased as extranodal extension grades increased. Recent studies demonstrated that high-grade radiologic extranodal extension with adjacent structure invasion significantly predicted a poor survival outcome [19–22]. However, the above studies did not eliminate the interference of confounding factors such as T stage, cervical lymph node level, laterality, and necrosis status. Thus, the reported prognostic value of radiologic characteristics in prior studies needs re-evaluation.

Additionally, accurate diagnosis of T3N1M0 patients is another key point. [^18^F]FDG PET/CT has advantages in detecting metastatic cervical lymph nodes and distant metastasis over MRI, but it is inferior in determining local tumor invasion and retropharyngeal nodal metastasis [23, 24]. This conclusion was based on the judgment of metastatic lymph nodes by clinical follow-up instead of pathologic confirmation. Herein, we included patients who underwent both MRI and [^18^F]FDG PET/CT examination before treatment. More importantly, one or more cervical lymph nodes of certain patients were histologically confirmed, so the performance of [^18^F]FDG PET/CT and MRI in diagnosing the specific lymph nodes provided firm evidence for precisely identifying the subgroup of T3N1M0. Subsequently, we accurately developed and validated the radiologic score of the lymph node characteristics to identify high-risk patients who can gain an additional survival benefit from induction chemotherapy and finally suggested an individualized treatment mode for these patients.

## Materials and methods

### Patients

**Fig. 1:**
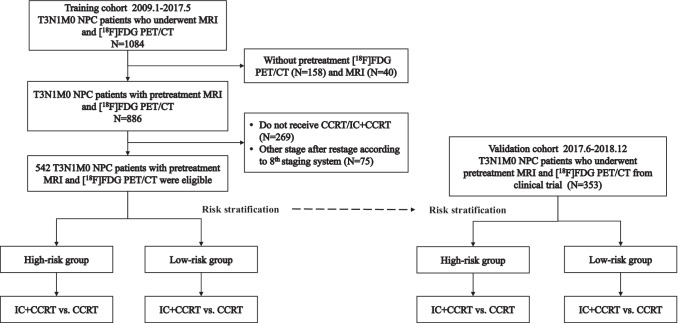
The flowchart of the included patients. CCRT, concurrent chemoradiotherapy; IC, induction chemotherapy; MRI, magnetic resonance imaging; NPC, nasopharyngeal carcinoma; [^18^F]FDG PET/CT, 2-deoxy-2-[^18^F]fluoro-d-glucose positron emission tomography/computed tomography

A training cohort of 542 patients staged with T3N1M0 nasopharyngeal carcinoma by pretreatment MRI and [^18^F]FDG PET/CT was obtained from May 2009 to May 2017 at Sun Yat-sen University Cancer Center. The eligibility criteria for selecting participants were as follows: (1) newly diagnosed nasopharyngeal carcinoma restaged as T3N1M0 in accordance with the 8th edition American Joint Committee on Cancer/Union for International Cancer Control (AJCC/UICC) staging system [25]; (2) receipt of pretreatment MRI and [^18^F]FDG PET/CT tests simultaneously; (3) receipt of concurrent chemoradiotherapy or induction followed by concurrent chemoradiotherapy; and (4) receipt of intensity-modulated radiotherapy. The patient flow chart is presented in Fig. 1.

A validation cohort containing 345 patients was obtained from our prospective clinical trial (ClinicalTrials.gov Identifier: NCT03003182) between June 2017 and September 2018.

This study was approved by the Institutional Ethical Review Board (No. B2021-059–01), and informed consent was waived for the part of retrospective analysis. Patients in the validation cohort were derived from a prospective observational study, and informed consent regarding a second analysis of their data was obtained from all of the patients.

### Image analysis

All patients received whole-body [^18^F]FDG PET/CT and MRI examinations of the head and neck. The detailed MRI and [^18^F]FDG PET/CT protocols are shown in the Supplementary Methods. All [^18^F]FDG PET/CT images were evaluated by a researcher (SSY, 5 years of experience in treating nasopharyngeal carcinoma) with reference to the issued report and then checked by an expert nuclear medicine physician (X.Z., more than 20 years of experience). All MR images were assessed by a radiation oncologist (P.Y.O.Y.) with 10 years of experience and reviewed again by an expert radiation oncologist (F.Y.X.) with over 30 years of experience in treating nasopharyngeal carcinoma. Inconsistencies were discussed with a radiologist (Y.H.) who had interpreted head and neck MR images of over 500 patients per month for over 5 years.

The diagnostic criteria for the metastatic lymph nodes, radiologic extranodal extension, and nodal necrosis were the same as those in previous studies [19, 26, 27] and are detailed in the Supplementary Methods. SUVmax was defined as the highest decay-corrected activity concentration per injected dose per body weight. The treatment and follow-up are shown in the Supplementary Methods.

### Statistical analysis

The primary endpoint was failure-free survival (FFS), which was defined as the time from diagnosis to failure (locoregional recurrence or distant metastasis) or death. The secondary endpoints were overall survival (OS, from diagnosis to death from any cause), regional relapse-free survival (RRFS, from diagnosis to regional recurrence or death), and distant metastasis-free survival (DMFS, from diagnosis to distant metastasis or death).

The sensitivity and specificity of [^18^F]FDG PET/CT and MRI were compared using McNemar’s paired-sample test, and confidence intervals for proportions were calculated according to the efficient-score method described by Robert Newcombe [28]. Time-dependent receiver operating characteristic (ROC) curve analysis was applied to determine the cutoff values of the continuous variables using the “survival ROC” package in R. The survival curves were compared by the log-rank test. Univariate and multivariate analyses were performed by Cox regression. Statistical analysis was conducted using SPSS 26.0 and R software (version 4.0.1, http://www.r-project.org/). A two-sided *p* < 0.05 was deemed statistically significant.

## Results

### Patient characteristics

After screening, 542 and 353 eligible patients were enrolled in the training cohort and the validation cohort, respectively. In the training cohort, the median age was 44 years, ranging from 16 to 73 years. The cutoff value of SUVmax-N was 9.3 for FFS (area under the curve [AUC]: 0.616, *p* = 0.001; Supplementary Fig. 2). The pretreatment EBV DNA cutoff point (2000 copies/mL) was determined according to previous studies and ROC curves. With a median follow-up time of 61 (range, 2–118) months for the 542 patients, 103 (19.0%) patients had treatment failure, and 38 (7.0%) patients suffered from regional recurrence. In addition, 7.4% (40/542) of patients had distant metastasis, while 6.6% (36/542) of patients died at the last follow-up. The 5-year FFS, RRFS, DMFS, and OS rates were 82.2%, 93.7%, 92.6%, and 94.5%, respectively. The baseline characteristics of the enrolled patients are shown in Table 1.

**Table 1:** Baseline characteristics in the training and validation cohorts

Characteristics	Number of patients (%)	
	Training cohort (*n* = 542)	Validation cohort (*n* = 353)
Median age (range)	44 (16–73)	48 (13–69)
< 52	399 (73.6)	230 (65.2)
≥ 52	143 (26.4)	123 (34.8)
Sex		
Male	381 (70.3)	247 (70.0)
Female	161 (29.7)	106 (30.0)
rENE		
Grade 0	260 (48.0)	204 (57.8)
Grade 1	107 (19.7)	62 (17.6)
Grade 2	111 (20.5)	58 (16.4)
Grade 3	64 (11.8)	29 (8.2)
Nodal necrosis		
Yes	93 (17.2)	54 (15.3)
No	449 (82.8)	299 (84.7)
SUVmax-N		
< 9.3	239 (44.1)	229 (64.9)
≥ 9.3	303 (55.9)	124 (35.1)
SUVmax-T		
< 16.3	455 (83.9)	279 (79.0)
≥ 16.3	87 (16.1)	74 (21.0)
Minimal axial diameter, median (range) cm	1.2 (0.3–4.1)	1.1 (0.4–3.0)
Maximal axial diameter, median (range) cm	1.7 (0.5–5.3)	1.1 (0.5–4.9)
Lymph node		
Retropharyngeal lymph node	92 (17.0)	92 (26.1)
Cervical lymph node	450 (83.0)	261 (73.9)
EBV DNA (copy/mL)		
< 2000	336 (62.0)	257 (72.8)
≥ 2000	206 (38.0)	96 (27.2)
Hemoglobin (g/L)		
< 120	18 (3.3)	14 (4.0)
≥ 120	524 (96.7)	339 (96.0)
LDH (U/L)		
< 250	520 (95.9)	344 (97.5)
≥ 250	22 (4.1)	9 (2.5)
Albumin (g/L)		
< 40	29 (5.4)	8 (2.3)
≥ 40	513 (94.7)	345 (97.7)
Treatment		
IC + CCRT	226 (41.7)	131 (37.1)
CCRT	316 (58.3)	222 (62.9)
IC regimen		
TPF	64 (11.8)	39 (11.0)
TP	82 (15.1)	53 (15.0)
PF	69 (12.7)	28 (7.9)
GP	11 (2.0)	11 (3.1)

*CCRT*, concurrent chemoradiotherapy; *EBV*, Epstein-Barr virus; *GP*, gemcitabine/cisplatin; *IC*, induction chemotherapy; *LDH*, serum lactate dehydrogenase; *PF*, cisplatin/5-fluorouracil; *rENE*, radiologic extranodal extension; *SUVmax-N*, the maximal standardized uptake value of lymph node; *SUVmax-T*, the maximal standardized uptake value of primary tumor; *TP*, docetaxel/cisplatin; *TPF*, docetaxel/cisplatin/5-fluorouracil

### [^18^F]FDG PET/CT versus MRI

In the whole cohort, 174 patients underwent cervical lymph node fine-needle aspiration biopsy guided by ultrasonography. Among the 224 biopsied lymph nodes of 174 patients, 132 and 92 lymph nodes were pathologically confirmed positive and negative, respectively. [^18^F]FDG PET/CT correctly diagnosed 129 positive lymph nodes and 74 negative lymph nodes, whereas MRI correctly found 115 positive lymph nodes and 59 negative lymph nodes. The sensitivity of [^18^F]FDG PET/CT (97.7%) was higher than that of MRI (87.1%) for detecting cervical lymph nodes (*p* < 0.001). The specificity, positive predictive value, and negative predictive value of [^18^F]FDG PET/CT and MRI were 80.4% vs. 64.1%, 87.8% vs. 77.7%, and 96.1% vs. 77.6%, respectively (see Table 2). As a result, 39 of 174 patients with lymph node biopsy showed an inconsistent N stage between [^18^F]FDG PET/CT and MRI. In the whole training cohort, 472 patients were consistently staged as T3 by [^18^F]FDG PET/CT or MRI; however, 20, 77, and 25 patients were possibly classified as N0, N2, or N3 by mistake if staged by MRI alone.

**Table 2:** Results of [^18^F]FDG PET/CT and MRI in detecting cervical lymph nodes confirmed by histopathology in 174 patients

	[^18^F]FDG PET/CT	MRI	*p*
TP	129	115	
TN	74	59	
FP	18	33	
FN	3	17	
Sensitivity (95% CI)	97.7 (93.0–99.4)	87.1 (79.9–92.1)	< 0.001
Specificity (95% CI)	80.4 (70.6–87.7)	64.1 (53.4–73.7)	< 0.001
PPV (95% CI)	87.8 (81.1–92.4)	77.7 (70.0–84.0)	0.022
NPV (95% CI)	96.1 (88.3–99.0)	77.6 (66.4–86.1)	< 0.001

*CI*, confidence interval; *FP*, false-positive; *FN*, false-negative; *NPV*, negative predictive value; *MRI*, magnetic resonance imaging; *[*^*18*^*F]FDG PET/CT*, 2-deoxy-2-[^18^F]fluoro-d-glucose positron emission tomography/computed tomography; *PPV*, positive predictive value; *TP*, true positive; *TN*, true negative

### Radiologic characteristics and survival outcomes

Of 542 patients, 93 (17.2%) patients had nodal necrosis. A total of 303 (55.9%) patients were assigned to the higher SUVmax-N group (≥ 9.3), and 87 (16.1%) patients had an SUVmax-T higher than 16.3. The median maximal axial diameter was 1.7 (range, 0.5–5.3) cm. The proportion of patients with grade 0, grade 1, grade 2, and grade 3 radiologic extranodal extension was 48.0% (260/542), 19.7% (107/542), 20.5% (111/542), and 11.8% (64/542), respectively, in the training cohort.

The analysis showed that patients with grade 3 radiologic extranodal extension had significantly lower FFS than those with grades 0, 1, and 2 radiologic extranodal extension (*p* < 0.001, *p* < 0.001, and *p* = 0.003). The survival curve is shown in Supplementary Fig. 3. As presented in Table 3, multivariate analysis demonstrated that SUVmax-N higher than 9.3, nodal necrosis, and grade 3 radiologic extranodal extension were independent factors of a poor prognosis for FFS (*p* = 0.035, *p* < 0.001, and *p* = 0.001, respectively). For graded radiologic extranodal extension, only grade 3, but not grade 0–2 radiologic extranodal extension, predicted an inferior FFS (hazard ratio [HR]: 2.703, 95% CI: 1.547–4.724, *p* = 0.001; Table 3).

**Table 3:** Univariate and multivariable analysis of FFS in the training cohort

Variable	Univariate analysis		Multivariable analysis	
	HR (95% CI)	*p*	HR (95% CI)	*p*
Age (≥ 52 vs. < 52)	1.145 (0.745–1.761)	0.537		
Sex (male vs. female)	0.879 (0.578–1.336)	0.546		
rENE				
Grade 0	Reference		Reference	
Grade 1	1.335 (0.762–2.341)	0.313	1.097 (0.617–1.952)	0.752
Grade 2	1.719 (1.019–2.899)	0.042	1.233 (0.705–2.157)	0.463
Grade 3	3.983 (2.373–6.685)	< 0.001	2.703 (1.547–4.724)	0.001
Nodal necrosis (yes vs. no)	2.544 (1.664–3.890)	< 0.001	1.897 (1.220–2.949)	< 0.001
SUVmax-N (≥ 9.3 vs. < 9.3)	2.240 (1.444–3.475)	< 0.001	1.672 (1.037–2.697)	0.035
SUVmax-T (≥ 16.3 vs. < 16.3)	1.145 (0.745–1.761)	0.456		
Minimal axial diameter	1.562 (1.201–2.031)	0.001		
Maximal axial diameter	1.353 (1.095–1.671)	0.005		
Lymph node (cervical lymph node vs. retropharyngeal lymph node)	2.139 (1.142–4.007)	0.018		
EBV DNA (≥ 2000 vs. < 2000)	1.340 (0.907–1.980)	0.141		
Hemoglobin (≥ 120 vs. < 120)	2.230 (0.533–9.328)	0.272		
LDH (≥ 250 vs. < 250)	0.748 (0.237–2.360)	0.620		
Albumin (≥ 40 vs. < 40)	0.535 (0.270–1.063)	0.074		
Treatment (IC + CCRT vs. CCRT)	0.728 (0.485–1.093)	0.126		

*CI*, confidence interval; *CCRT*, concurrent chemoradiotherapy; *EBV*, Epstein-Barr virus; *FFS*, failure-free survival; *HR*, hazard ratio; *IC*, induction chemotherapy; *LDH*, serum lactate dehydrogenase; *rENE*, radiologic extranodal extension; *SUVmax-N*, the maximal standardized uptake value of lymph node; *SUVmax-T*, the maximal standardized uptake value of primary tumor

Similarly, the above lymph node characteristics were also independent factors for DMFS, while grade 3 radiologic extranodal extension was the only significant independent factor for RRFS (Supplementary Table 1).

### Radiologic score and risk stratification

**Fig. 2:**
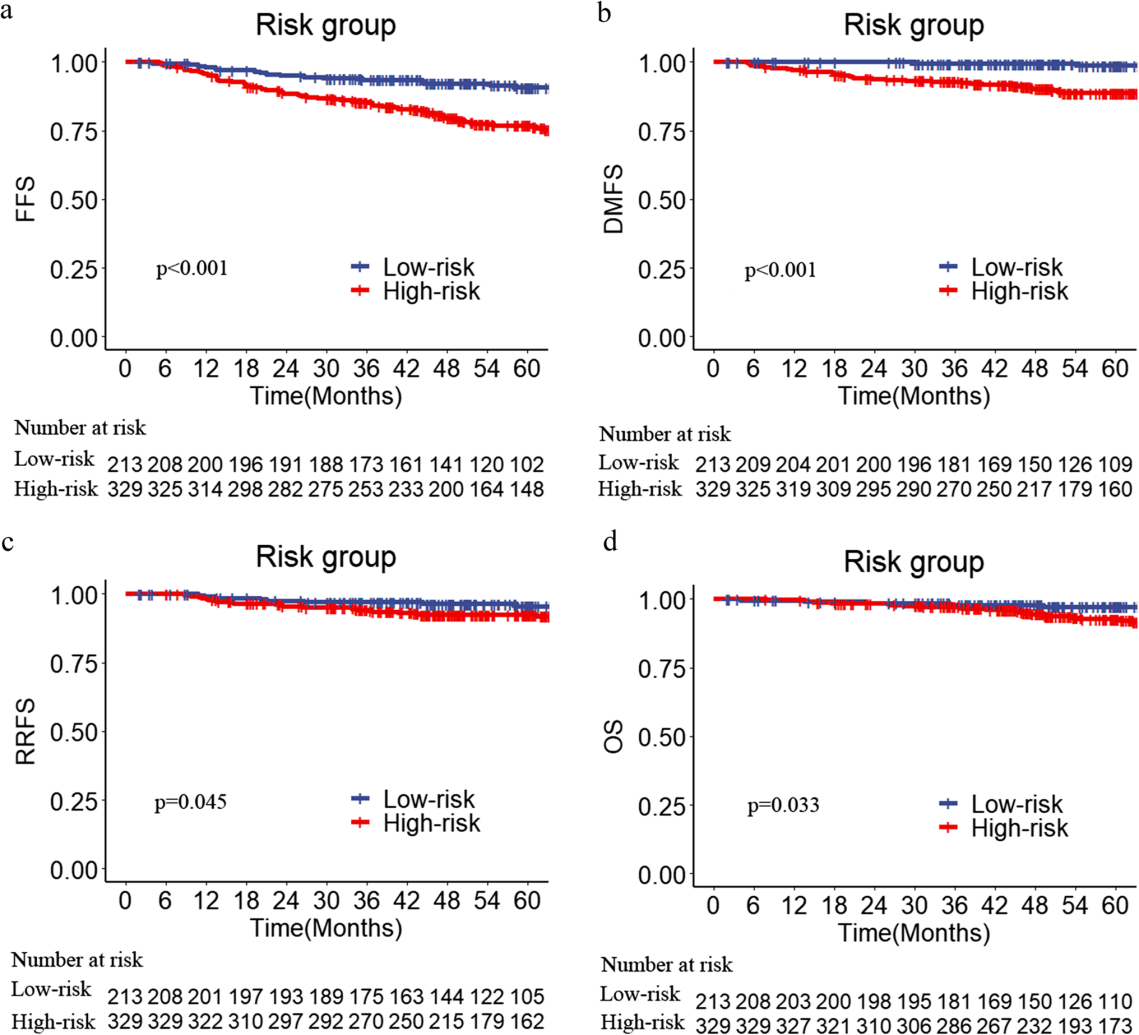
Survival curves of high- and low-risk groups stratified by radiologic score in FFS (**a**), DMFS (**b**), RRFS (**c**), and OS (**d**) in the training cohort. High-risk group: radiologic score > 0, low-risk group: radiologic score = 0. DMFS, distant metastasis-free survival; FFS, failure-free survival; OS, overall survival; RRFS, regional relapse-free survival

Prognostic factors obtained from the multivariate analysis were used for risk stratification. One risk factor scored 1 point, and patients were thus scaled from 0 to 3 points according to the number of risk factors. The survival curve revealed that patients with higher radiologic scores had lower survival rates (*p* < 0.001; Supplementary Fig. 4). Therefore, patients were stratified into a high-risk group (radiologic score > 0, *n* = 329) and a low-risk group (radiologic score = 0, *n* = 213) by their radiologic score. The baseline characteristics of participants in both risk groups are summarized in Supplementary Table 2. As shown in Fig. 2, the 5-year FFS, DMFS, RRFS, and OS rates were 90.7% vs. 77.0%, 98.7% vs. 88.8%, 95.5% vs. 92.5%, and 97.3% vs. 92.8% for patients in the low-risk and high-risk groups, respectively (all *p* < 0.05).

### Benefit of induction chemotherapy

**Fig. 3:**
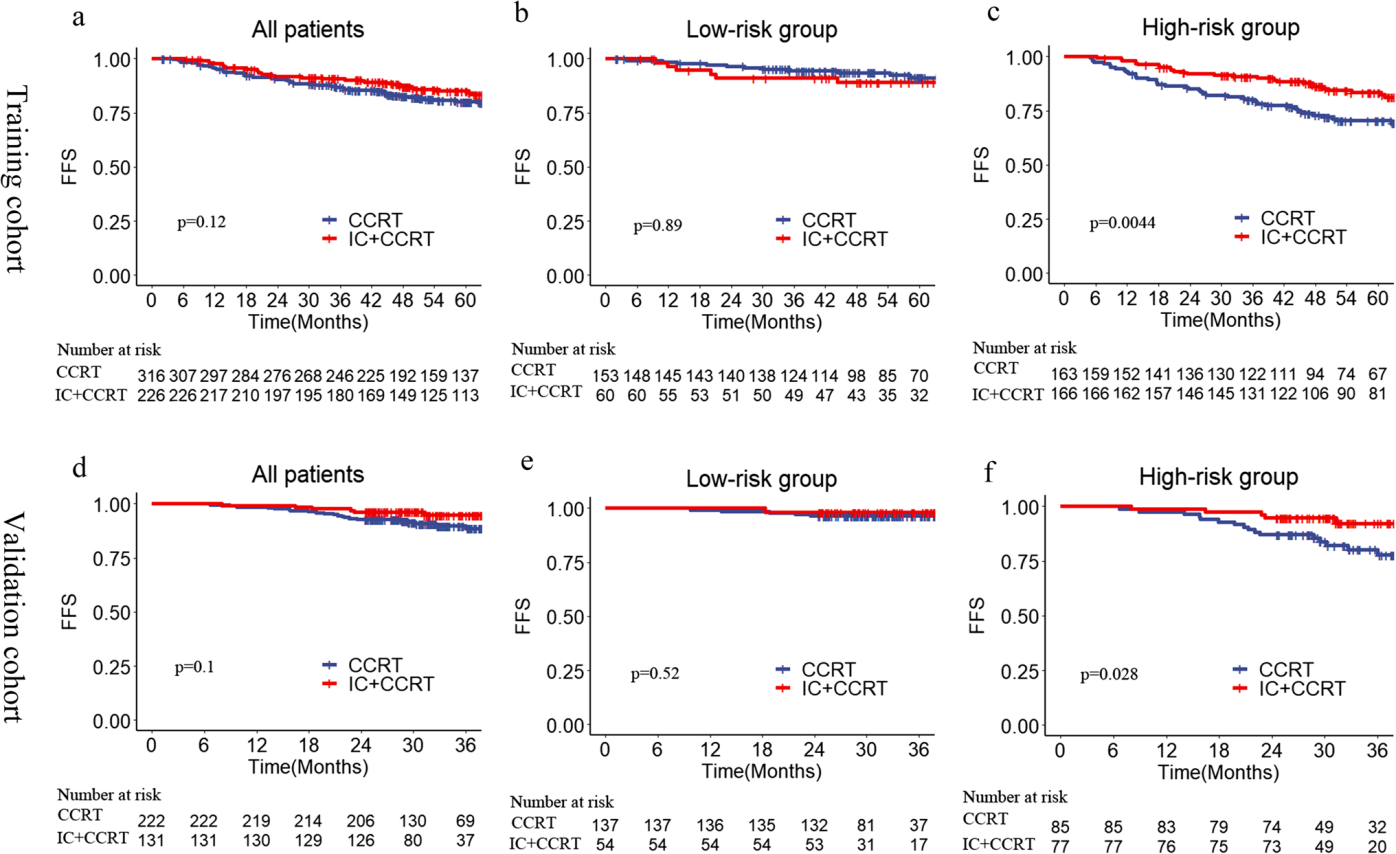
Kaplan–Meier FFS curves of IC + CCRT and CCRT alone in the training cohort (whole cohort (**a**), low-risk group (**b**), high-risk group (**c**)) and validation cohort (whole cohort (**d**), low-risk group (**e**), high-risk group (**f**)). CCRT, concurrent chemoradiotherapy; FFS, failure-free survival; IC, induction chemotherapy

In the whole training cohort, FFS was not significantly different for patients with or without induction chemotherapy before concurrent chemoradiotherapy (HR: 0.72, 95% CI: 0.49–1.09, *p* = 0.12 by univariate analysis; Fig. 3a). However, in the high-risk group, patients who received induction followed by concurrent chemoradiotherapy had better FFS than patients who received concurrent chemoradiotherapy alone (5-year FFS: 83.5% vs. 70.5%, *p* = 0.0044; Fig. 3c). Additionally, multivariate Cox regression analysis indicated that induction chemotherapy plus concurrent chemoradiotherapy was still an independent prognostic factor for FFS (HR: 0.48, 95% CI: 0.305–0.755, *p* = 0.002; Table 4) in the high-risk group. In the low-risk group, similar survival outcomes were observed between induction chemotherapy followed by concurrent chemoradiotherapy and concurrent chemoradiotherapy alone (5-year FFS: 91.3% vs. 89.1%, *p* = 0.89; Fig. 3).

**Table 4:** Results of multivariate analysis for the high-risk group in the training cohort

Endpoints	Variables	HR (95% CI)	*p*
FFS	rENE (grade 3 vs. grades 0–2)	2.508 (1.557–4.040)	< 0.001
	Nodal necrosis (yes vs. no)	1.780 (1.133–2.795)	0.012
	Treatment (IC + CCRT vs. CCRT)	0.480 (0.305–0.755)	0.002
DMFS	rENE (grade 3 vs. grades 0–2)	2.046 (1.004–4.173)	0.045
	Nodal necrosis (yes vs. no)	2.085 (1.081–4.021)	0.028
	Treatment (IC + CCRT vs. CCRT)	0.460 (0.234–0.904)	0.024
RRFS	Nodal necrosis (yes vs. no)	2.461 (1.115–5.429)	0.026
	Treatment (IC + CCRT vs. CCRT)	0.409 (0.186–0.902)	0.027
OS	Age (≥ 52 vs. < 52)	2.546 (1.213–5.346)	0.014
	Albumin (≥ 40 vs. < 40)	0.328 (0.125–0.863)	0.024

*CI*, confidence interval; *CCRT*, concurrent chemoradiotherapy; *DMFS*, distant metastasis-free survival; *FFS*, failure-free survival; *HR*, hazard ratio; *IC*, induction chemotherapy; *OS*, overall survival; *rENE*, radiologic extranodal extension; *RRFS*, regional relapse-free survival

The results for DMFS and RRFS are shown in Supplementary Fig. 5, Supplementary Table 3, and Table 4.

### Validation

As shown in Table 1, the median age was 48 years (range, 13–69) in the validation cohort. With a median follow-up time of 32 (range, 14–44) months for these 353 patients, 27 developed treatment failure. The 3-year FFS and RRFS were 90.8% and 93.7%, respectively. Patients with nodal necrosis accounted for 15.3%, and 124 patients had a higher SUVmax-N (≥ 9.3). Among 353 patients, 204 (57.8%), 62 (17.6%), 58 (16.4%), and 29 (8.2%) patients had grade 0, grade 1, grade 2, and grade 3 radiologic extranodal extension, respectively. Grade 3 radiologic extranodal extension, SUVmax-N (≥ 9.3), and nodal necrosis were confirmed as significant factors of a poor prognosis for FFS in the validation group (all *p* < 0.05; Supplementary Fig. 6).

The prospective validation set was stratified into a high-risk group (*n* = 162) and a low-risk group (*n* = 191) according to the radiologic score identified in the training set. The 3-year FFS rate for patients in the high-risk group was lower than that for patients in the low-risk group (96.9% vs. 84.2%, *p* < 0.001; Supplementary Fig. 6e).

As shown in Fig. 3, there were no significant differences in FFS between the two treatment models in the whole validation cohort and the low-risk group (*p* = 0.1, *p* = 0.52). However, patients undergoing induction chemotherapy followed by concurrent chemoradiotherapy had a higher survival rate than those undergoing concurrent chemoradiotherapy alone in the high-risk group (3-year FFS: 92.2% vs. 80.2%; HR: 0.34, 95% CI: 0.13–0.93, *p* = 0.028; Fig. 3f).

## Discussion

In this large cohort study, [^18^F]FDG PET/CT was more accurate than MRI for detecting metastatic cervical lymph nodes, which provided firm evidence for precisely identifying T3N1M0 patients by both [^18^F]FDG PET/CT and MRI. Radiologic lymph node characteristics, including SUVmax-N higher than 9.3, nodal necrosis, and grade 3 radiologic extranodal extension, were independent prognostic factors for nasopharyngeal carcinoma patients staged as T3N1M0. Accordingly, high-risk and low-risk groups could be stratified by these risk factors instead of sex and EBV DNA load. We demonstrated that only patients in the high-risk group could benefit from the addition of induction chemotherapy. These findings were verified again by the validation cohort from our clinical trial.

In this study, EBV DNA load was not confirmed for separating the subgroup of T3N1M0. As 206 and 96 patients in the training and validation cohorts had EBV DNA loads higher than 2000 copies/mL, an insufficient sample size may not justify this result. Similarly, female patients also did not show a superior survival rate. Consistent with a prior study [29], [^18^F]FDG PET/CT did have better diagnostic accuracy than conventional imaging in nasopharyngeal carcinoma, as proven by the histological results of the lymph nodes. Therefore, the subgroup of T3N1M0 staged by both [^18^F]FDG PET/CT and MRI in this study showed a relatively high survival rate, which was close to the reported rate of stage II patients [30]. As a result, extremely strong predictive markers are required for further separation of this subgroup.

[^18^F]FDG PET/CT, as functional imaging, provides metabolic information and can guide prognostication [31]. Prior studies have reported that SUVmax-T, SUVmax-N, and SUV_75%_ of primary tumors are prognostic factors for nasopharyngeal carcinoma [32, 33]. Thus, it was not absurd that SUVmax-N also acted as an independent prognostic factor in the subgroup of patients with T3N1M0. Pathologic extranodal extension has been introduced into the N classification for nonviral-related head and neck cancer in the 8th edition of the AJCC/UICC staging system. Due to the radiotherapy-based primary treatment, pathologic extranodal extension is not available for nasopharyngeal carcinoma. However, radiologic extranodal extension based on MRI or CT has good specificity and sensitivity (ranging from 70 to 90%) in predicting pathologic extranodal extension in head and neck cancer [34]. The specificity of radiologic extranodal extension infiltrating adjacent structures is nearly 100%, consistent with pathologic extranodal extension [18, 35]. Therefore, radiologic extranodal extension based on MRI is an accepted surrogate of pathologic extranodal extension for nasopharyngeal carcinoma. Similar to previous studies [19–21], the most severe radiologic extranodal extension with the involvement of adjacent structures was correlated with poor survival outcomes in the subgroup of T3N1M0 nasopharyngeal carcinoma. In addition, nodal necrosis, a vital radiologic nodal feature, is a reliable sign for detecting nodal metastasis. MRI has similar sensitivity to CT in identifying nodal necrosis [27]. Previous studies indicated that nodal necrosis is a strong prognostic factor in nasopharyngeal carcinoma, as the survival rate of patients with nodal necrosis declined nearly 12% in comparison with that of patients without nodal necrosis [36]. Therefore, it was not unreasonable that nodal necrosis was a significant predictor of outcomes in the T3N1M0 subgroup.

In previous studies [19–21], the TNM stage of enrolled patients varied from stage I to stage IVa, which contained significant heterogeneity. The confounding factors, including T stage, nodal size, nodal level, nodal laterality, and treatment modes, could not be completely eliminated. In our study, the metastatic lymph nodes of T3N1M0 patients were located in the unilateral upper cervical region, which fully controlled the covariate factors of T stage and radiological lymph node characteristics, such as nodal level and nodal laterality. As mentioned above, after eliminating confounding factors, our study confirmed that the three radiological lymph node characteristics, namely SUVmax-N, extranodal extension, and nodal necrosis, were closely related to survival outcome, especially distant metastasis. Perhaps if lymph nodes have a high metabolic rate and tumors spread outside the nodal capsule, tumor cells can easily enter the blood circulation and finally develop metastasis in distant organs. Although intensity-modulated radiotherapy delivers radical doses to the primary tumor and metastatic lymph nodes and can achieve excellent locoregional control [4], the high risk of distant metastasis for patients with these sorts of nodal characteristics cannot be reduced. As we found in the present study, high-risk T3N1M0 patients had a similar 5-year FFS rate to patients who were enrolled in clinical trials of induction chemotherapy [37]. Given the confirmed benefit of induction chemotherapy in locoregionally advanced nasopharyngeal carcinoma from randomized controlled trials [5–7] and meta-analyses [38, 39], it is highly reasonable that induction chemotherapy plus concurrent chemoradiotherapy can improve the survival rate of high-risk T3N1M0 patients.

There are several advantages of this study. First, all patients were restaged by [^18^F]FDG PET/CT and MRI according to the 8th AJCC/UICC staging system. The subset of patients who underwent biopsy of certain cervical lymph nodes demonstrated the accuracy of [^18^F]FDG PET/CT in detecting positive lymph nodes, which supported the reliability of the T3N1M0 staging of the patients. Notably, all eligible patients were upper cervical lymph node positive and unilateral lymph node positive, which fully eliminated covariate factors, including T stage, nodal laterality, nodal level, and nodal size. In addition, the sample size was relatively large, and the results were verified by a validation cohort. Limitations of this study should also be noted. First, this was a single-center study, and WHO type III was the predominant pathology type. Second, the follow-up duration of the validation cohort was not long enough. Hence, subsequent follow-up is warranted.

## Conclusion

In conclusion, T3N1M0 patients could be diagnosed more accurately by both [^18^F]FDG PET/CT and MRI. The radiologic score of lymph node characteristics based on MRI and [^18^F]FDG PET/CT could correctly identify high-risk patients who can obtain additional survival benefit from induction chemotherapy and it could be used to guide individualized treatment for nasopharyngeal carcinoma patients staged with T3N1M0 in clinical practice.

## Supplementary Information

Below is the link to the electronic supplementary material.

Supplementary file1 (PDF 2.87 MB)

## Abbreviations

[^18^F]FDG PET/CT: 2-Deoxy-2-[^18^F]fluoro-d-glucose positron emission tomography/computed tomography
AJCC/UICC: American Joint Committee on Cancer/Union for International Cancer Control
AUC: Area under the curve
CCRT: Concurrent chemoradiotherapy
CI: Confidence interval
CTV: Clinical target volume
DMFS: Distant metastasis-free survival
EBV: Epstein-Barr virus
FFS: Failure-free survival
FN: False-negative
FP: False-positive
GP: Gemcitabine/cisplatin
HR: Hazard ratio
IC: Induction chemotherapy
K-M: Kaplan–Meier
LDH: Serum lactate dehydrogenase
MRI: Magnetic resonance imaging
NCCN: National Comprehensive Cancer Network
NPC: Nasopharyngeal carcinoma
NPV: Negative predictive value
OS: Overall survival
OSEM: Ordered subset expectation maximization
PF: Cisplatin/5-fluorouracil
PPV: Positive predictive value
rENE: Radiologic extranodal extension
ROC: Receiver operating characteristic
RRFS: Regional relapse-free survival
SUVmax-N: The maximal standardized uptake value of lymph node
SUVmax-T: The maximal standardized uptake value of the primary tumor
TN: True negative
TP: Docetaxel/cisplatin
TP: True positive
TPF: Docetaxel/cisplatin/5-fluorouracil

## Declarations

### Guarantor

The scientific guarantor of this publication is Fang-Yun Xie.

### Statistics and biometry

No complex statistical methods were necessary for this paper.

### Methodology

• retrospective plus prospective validation

• observational

• performed at one institution
