# Supplementary material for: Development and validation of radiologic scores for guiding individualized induction chemotherapy in T3N1M0 nasopharyngeal carcinoma
Source: Eur Radiol. 2022 Jan 6;32(6):3649–60. doi: 10.1007/s00330-021-08460-1 (PMC9123027; doi:10.1007/s00330-021-08460-1)
Supplement: Supplementary file 1 — Supplementary file1 (PDF 2.87 MB) [file 330_2021_8460_MOESM1_ESM.pdf]

Supplementary Method

MRI protocol

All patients underwent head and neck MR imaging with a 1.5- or 3.0-T system (Signa CV/i, GE HealthCare). The area from the suprasellar cistern to the inferior margin of the sternal end of the clavicle was scanned. T1-weighted fast spin-echo images in the axial, coronal, and sagittal planes (repetition time: 500–600 ms, echo time: 10–20 ms, and field of view: 22 cm) and T2-weighted fast spin-echo MR images in the axial plane (repetition time: 4000–6000 ms, echo time: 95–110 ms, and field of view: 22 cm) were obtained before injection of contrast material. Spin-echo T1-weighted axial and sagittal sequences and spin-echo T1-weighted fat-suppressed coronal sequences were performed after intravenous Gd-DTPA (Magnevist; Bayer Schering Pharma AG) injection at a dose of 0.1 mmol/kg.

[^{18}F]FDG PET/CT protocol

[^{18}F]FDG PET/CT scans were conducted using a Discovery ST-16 (GE Medical Systems, Milwaukee). The scan range was from the vertex to the upper thigh according to a standard whole-body acquisition protocol. Patients needed to fast for 6 hours before injecting [^{18}F]FDG. Then, imaging was performed approximately 45–60 mins after injection of 3.7 Mbq/kg of body weight (0.1 mCi/kg) of [^{18}F]FDG. Next, a low-dose multislice CT scan was obtained using a 16-slice multidetector scanner (parameters: 180–250 mA, 140 kV, pitch 1.375 mm, and slice thickness 3.75 mm) with shallow breathing. A standard whole-body PET scan was required in 2D mode with an acquisition time of 3 mins per bed position (six-eight bed positions) covering the same field as the CT scan. The acquired data were reconstructed using the ordered subset expectation maximization (OSEM) iterative algorithm. Finally, the data were transferred to a workstation (AW Server 2.0; GE Health care) for processing and interpretation. The standardized uptake value (SUV) was calculated using the body weight.

Radiologic criteria

The radiologic criteria of the metastatic lymph nodes were as follows: (1) minimal axial diameter of 5 mm and 10 mm for retropharyngeal lymph nodes and cervical lymph nodes, respectively; (2) clusters of 3 or more lymph nodes with a minimal axial diameter of 8 mm; and (3) nodal necrosis or extranodal extension. Similar to a recent study, radiologic extranodal extension was categorized into 4 grades: Grade 0, no extranodal extension; Grade 1, invasion to surrounding fat; Grade 2, coalescent nodes; and Grade 3, infiltrating adjacent structures. As reported, the diagnostic criteria for nodal necrosis based on MRI included (1) focal area of high signal intensity on T2-weighted images and (2) focal area of low signal intensity on T1-weighted images with or without enhanced edges **(Supplementary Figure 1)**.

Treatment and follow-up

All participants were treated with radical intensity-modulated radiotherapy. Delineation of the target volumes was consistent with the International Commission on Radiation Units and Measurements Reports 50 and 62. The prescribed doses were 66–72 Gy/28–33 fractions to the gross tumour and 64–70 Gy/28–33 fractions to the lymph nodes, 60–63 Gy/28–33 fractions to the high-risk clinical target volume (clinical target volume, CTV1), and 54–56 Gy/28–33 fractions to the low-risk clinical target volume (CTV2).

Induction chemotherapy regimens included docetaxel/cisplatin/5-fluorouracil (TPF, 60 mg/m2, 60 mg/m2, and 3,000 mg/m2, respectively), cisplatin/5-fluorouracil (PF, 80 mg/m2 and 4,000 mg/m2, respectively), and docetaxel/cisplatin (TP, 75 mg/m2 and 75 mg/m2) every 3 weeks for 2–4 cycles. For concurrent chemotherapy, weekly cisplatin (30–40 mg/m2) or a 3-weekly cisplatin (80–100 mg/m2)

regimen was administered.

After treatment, follow-up was performed every 3 to 6 months during the first 2 years and then every 6 to 12 months thereafter. Routine evaluation measures included head and neck MRI, nasopharyngoscopy, chest X-ray or computed tomography, and abdominal sonography or computed tomography. [^{18}F]FDG PET/CT was performed if necessary, and a biopsy was conducted to confirm recurrence or metastasis if possible.

Supplementary Table 1. Univariate analysis of DMFS and RRFS in the training cohort.

	DMFS				RRFS			
	Univariate analysis		Multivariable analysis		Univariate analysis		Multivariable analysis	
Variable	HR (95% CI)	*p*	HR (95% CI)	*p*	HR (95% CI)	*p*	HR (95% CI)	*p*
Age (≥ 52 vs. < 52)	0.947 (0.463-1.938)	0.882			0.553 (0.170-1.799)	0.325		
Sex (Male vs. Female）	1.109 (0.550-2.220)	0.769			0.617 (0.322-1.182)	0.146		
rENE (Grade 3 vs. Grade 0-2)	3.450 (1.720-6.940)	0.001	2.123 (1.040-4.336)	0.039	2.776 (1.312-5.874)	0.008	1.400 (1.000-2.000)	0.038
Nodal Necrosis (Yes vs. No)	3.810 (2.010-7.240)	< 0.001	2.579 (1.338-4.971)	0.005	2.164 (1.051-4.456)	0.036		
SUVmax-N (≥ 9.3 vs. < 9.3)	5.487 (2.150-14.006)	< 0.001	4.082 (1.571-10.611)	0.004	0.604 (0.214-1.703)	0.340		
SUVmax-T (≥ 16.3 vs. < 16.3)	1.539 (0.732-3.235)	0.255			1.203 (0.597-2.426)	0.605		
Minimal axial diameter	1.890 (1.280-2.810)	0.002			1.773 (1.164-2.701)	0.008		
Maximal axial diameter	1.657 (1.210-2.275)	0.002			1.471 (1.045-2.071)	0.027		
Lymph Node (Retropharyngeal lymph node vs. Cervical lymph node)	0.214 (0.05-0.888)	0.034			0.3490 (0.107-1.138)	0.081		
EBV DNA (≥ 2000 vs. < 2000)	1.340 (0.907-1.980)	0.141			1.347 (0.710-2.560)	0.362		
Hemoglobin (≥ 120 vs. < 120)	21.240 (0.010-36036)	0.421			0.639 (0.154-2.660)	0.538		
LDH (≥ 250 vs. < 250)	0.679 (0.090-4.946)	0.702			0.047 (0-80.790)	0.421		
Albumin (≥ 40 vs. < 40)	0.637 (0.200-2.069)	0.453			0.578 (0.177-1.885)	0.363		
Treatment (IC+CCRT vs. CCRT)	0.644 (0.330-1.249)	0.193			0.806 (0.417-1.559)	0.522		

Abbreviations: CI, confidence interval; CCRT, concurrent chemoradiotherapy; DMFS, distant metastasis-free survival; EBV, Epstein-Barr virus; HR, hazard ratio; IC, induction chemotherapy; LDH, serum lactate dehydrogenase; RRFS, regional relapse-free survival; rENE, radiologic extranodal extension; SUVmax-N, the maximal standardized uptake value of lymph node; SUVmax-T, the maximal standardized uptake value of primary tumor.

Supplementary Table 2. The baseline characteristics of the different risk groups in the training cohort.

		Number of patients (%)	
		High-risk group (n=329)	Low-risk group (n=213)
Age			
Median (range)		44 (19-73)	45 (16-71)
	< 52	240 (72.9)	159 (74.6)
	≥ 52	89 (27.1)	54 (25.4)
Sex			
	Male	97 (29.5)	64 (30.0)
	Female	232 (70.5)	149 (70.0)
SUVmax-T			
	< 16.3	242 (73.6)	213 (100.0)
	≥ 16.3	87 (26.4)	0 (0.0)
SUVmax-N			
	< 9.3	26 (7.9)	213 (100.0)
	≥ 9.3	303 (92.1)	0 (0.0)
Maximal axial diameter			
Median (range)		2.1 (0.7-5.3)	1.2 (0.5-4.2)
Minimal axial diameter			
Median (range)		1.5 (0.5-4.1)	0.8 (0.3-2.6)
Hemoglobin (g/L)			
	< 120	11 (3.3)	7 (3.3)
	≥ 120	318 (96.7)	206 (96.7)
Albumin (g/L)			
	< 40	22 (6.7)	7 (3.3)

≥ 40	307 (93.3)	206 (96.7)
LDH(U/L)		
< 250	312 (94.8)	208 (97.7)
≥ 250	17 (5.2)	5 (2.3)
EBV DNA (copy/mL)		
< 2000	164 (49.8)	172 (80.8)
≥ 2000	165 (50.2)	41 (19.2)
rENE		
Grade 0	107 (32.5)	153 (71.8)
Grade 1	67 (20.4)	40 (18.8)
Grade 2	91 (27.7)	20 (9.4)
Grade 3	64 (19.5)	0 (0.0)
Nodal Necrosis		
Yes	236 (71.7)	213 (100.0)
No	93 (28.3)	0 (0.0)
Lymph Node		
Retropharyngeal lymph node	297 (90.3)	153 (71.8)
Cervical lymph node	32 (9.7)	60 (28.2)
Treatment		
CCRT	163 (49.5)	153 (71.8)
IC + CCRT	166 (50.5)	60 (28.2)
IC regimen		
TPF	47 (14.3)	17 (8.0)
TP	60 (18.2)	22 (10.3)
PF	52 (15.8)	17 (8.0)
GP	7 (2.1)	4 (1.9)

Abbreviations: CCRT, concurrent chemoradiotherapy; EBV, Epstein-Barr virus; GP: gemcitabine-cisplatin; IC, induction chemotherapy; LDH, serum lactate dehydrogenase; PF, cisplatin/5-fluorouracil; rENE, radiologic extranodal extension; SUVmax-N, the maximal standardized uptake value of lymph node; SUVmax-T, the maximal standardized uptake value of primary tumor; TP, docetaxel/cisplatin; TPF, docetaxel/cisplatin/5-fluorouracil.

Supplementary Table 3. Univariate analysis of the high-risk group in the training cohort.

	FFS		DMFS		RRFS		OS	
	HR (95% CI)	*p*	HR (95% CI)	*p*	HR (95% CI)	*p*	HR (95% CI)	*p*
Sex (Male vs. Female)	0.939 (0.587-1.504)	0.795	1.050 (0.520-2.117)	0.892	0.785 (0.365-1.688)	0.535	1.347 (0.573-3.170)	0.495
Age (≥ 52 vs. < 52)	1.299 (0.819-2.060)	0.266	0.917 (0.445-1.890)	0.814	0.881 (0.376-2.064)	0.771	2.579 (1.229-5.414)	0.012
SUVmax-T (≥ 16.3 vs. < 16.3)	0.826 (0.494-1.379)	0.464	0.857 (0.405-1.812)	0.686	0.433 (0.151-1.243)	0.12	1.288 (0.583-2.848)	0.531
SUVmax-N (≥ 9.3 vs. < 9.3)	0.844 (0.366-1.946)	0.691	0.794 (0.243-2.600)	0.704	1.982 (0.268-14.652)	0.503	0.435 (0.128-1.475)	0.182
Maximal axial diameter	1.043 (0.794-1.372)	0.761	1.181 (0.800-1.742)	0.403	1.181 (0.753-1.852)	0.468	0.744 (0.451-1.228)	0.248
Minimal axial diameter	1.123 (0.798-1.579)	0.506	1.191 (0.728-1.948)	0.487	1.362 (0.786-2.359)	0.271	0.814 (0.433-1.528)	0.521
Albumin (≥ 40 vs. < 40)	0.669 (0.322-1.387)	0.280	0.790 (0.243-2.574)	0.696	0.594 (0.180-1.964)	0.394	0.320 (0.122-0.841)	0.021
LDH (≥ 250 vs. < 250)	0.420 (0.103-1.710)	0.226	0.496 (0.068-3.621)	0.490	0.046 (0-60.445)	0.400	1.382 (0.328-5.827)	0.659
EBV DNA (≥ 2000 vs. < 2000)	0.890 (0.576-1.374)	0.598	0.672 (0.349-1.295)	0.235	0.829 (0.398-1.727)	0.616	1.509 (0.706-3.225)	0.288
rENE (Grade 3 vs. Grade 0-2)	2.415 (1.507-3.872)	< 0.001	2.050 (1.012-4.153)	0.046	2.256 (1.026-4.965)	0.043	1.946 (0.857-4.422)	0.112
Nodal Necrosis (Yes vs. No)	1.866 (1.190-2.926)	0.007	2.188 (1.137-4.209)	0.019	1.286 (0.582-2.840)	0.534	1.187 (0.520-2.709)	0.683
Lymph Node (Retropharyngeal lymph node vs. Cervical lymph node)	0.437 (0.177-1.083)	0.074	0.199 (0.027-1.453)	0.111	0.249 (0.034-1.837)	0.173	0.791 (0.238-2.629)	0.702
Treatment (IC+CCRT vs. CCRT)	0.526 (0.336-0.825)	0.005	0.491 (0.251-0.962)	0.038	0.437 (0.199-0.960)	0.039	1.170 (0.557-2.460)	0.678

Abbreviations: CI, confidence interval; CCRT, concurrent chemoradiotherapy; DMFS, distant metastasis-free survival; EBV, Epstein-Barr virus; FFS, failure-free survival; HR, hazard ratio; IC, induction chemotherapy; LDH, serum lactate dehydrogenase; OS, overall survival; RRFS, regional relapse-free survival; rENE, radiologic extranodal extension; SUVmax-N, the maximal standardized uptake value of lymph node; SUVmax-T, the maximal standardized uptake value of primary tumor.

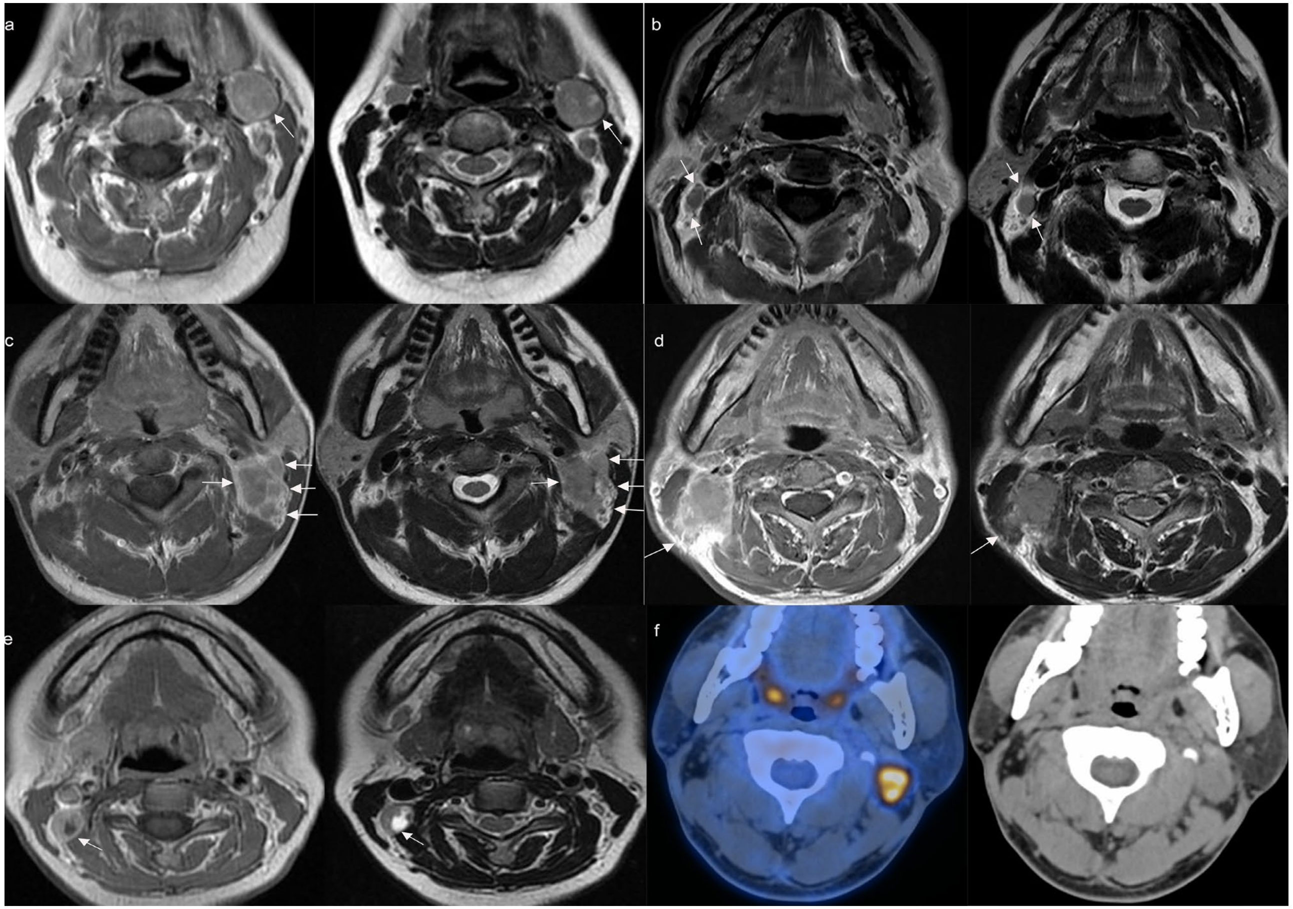

Supplementary Figure 1. Axial T1-weighted fat-suppressed contrast-enhanced (left) and T2-weighted (right) MRI. (a) Grade 0 rENE; (b) grade 1 rENE; (c) grade 2 rENE; (d) grade 3 rENE; (e) nodal necrosis; (f) [^{18}F]FDG PET/CT.

Abbreviations: MRI, magnetic resonance imaging; rENE, radiologic extranodal extension; [^{18}F]FDG PET/CT, 2-deoxy-2-[^{18}F]fluoro-D-glucose positron emission tomography/computed tomography.

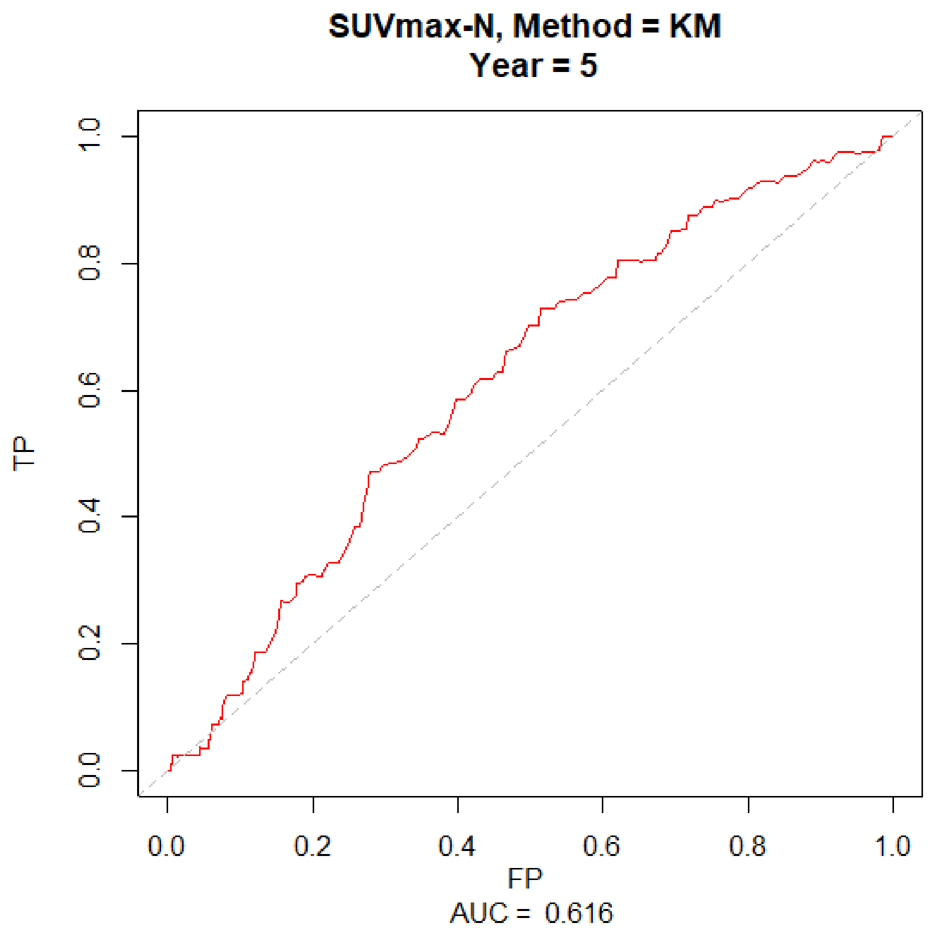

Supplementary Figure 2 Time-dependent receiver operating characteristic (ROC) curve of SUVmax-N

Abbreviations: AUC, area under the curve; FP, false positive; K-M, Kaplan-Meier; SUVmax-N, the maximal standardized uptake value of lymph node; TP, true positive.

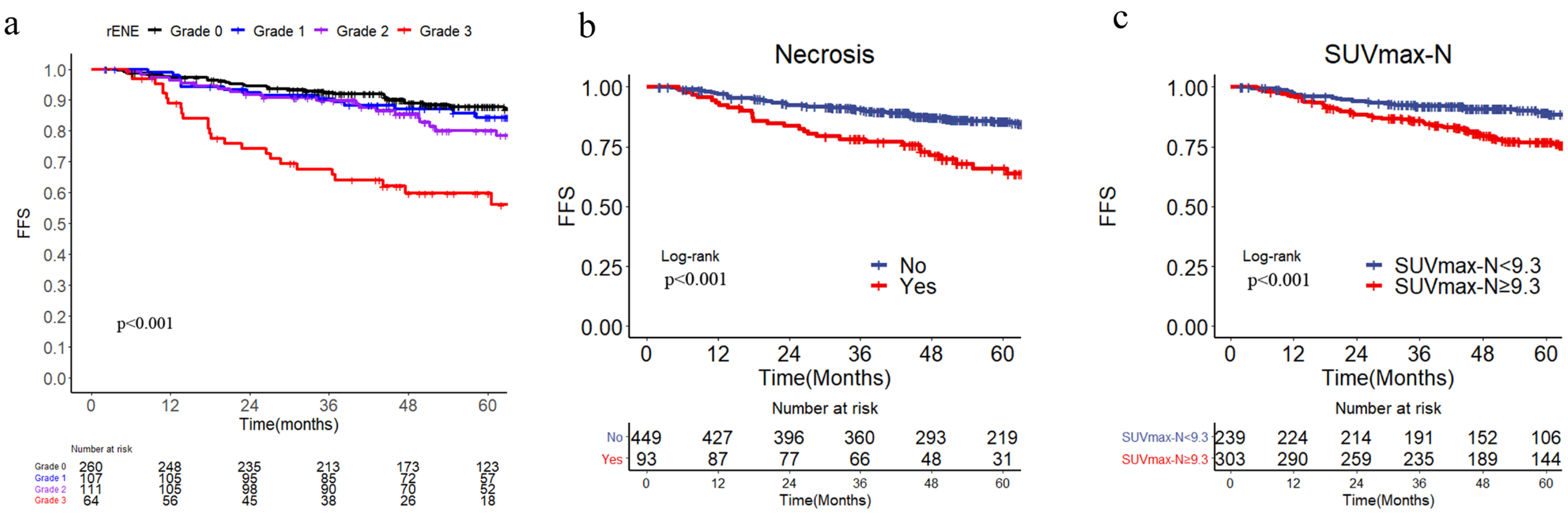

Supplementary Figure 3 Kaplan–Meier survival curves of rENE (a), necrosis (b) and SUVmax-N (c) for FFS in the whole training cohort

Abbreviations: FFS, failure-free survival; rENE, radiologic extranodal extension; SUVmax-N, the maximal standardized uptake value of lymph node.

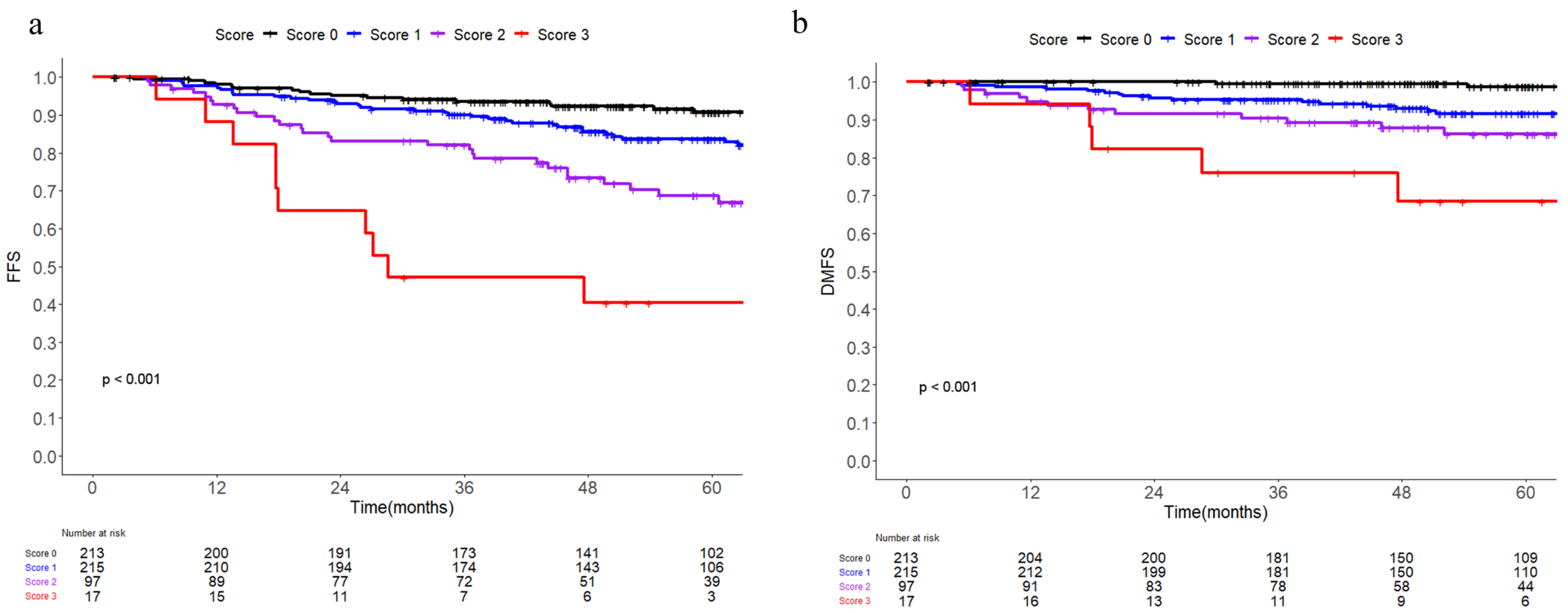

Supplementary Figure 4 Survival curves of risk scores for FFS (a) and DMFS (b) in the training cohort

Abbreviations: DMFS, distant metastasis-free survival; FFS, failure-free survival.

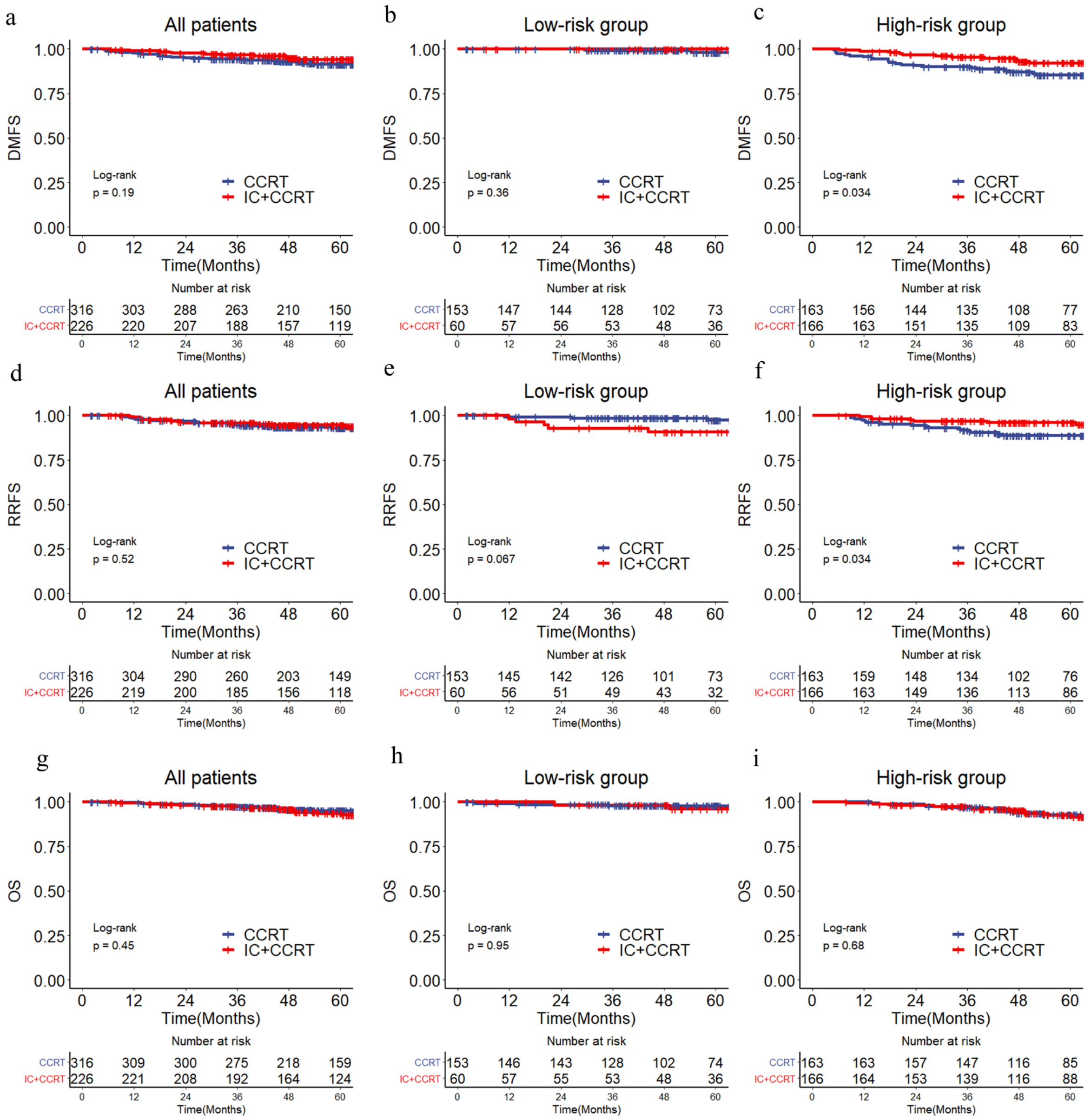

Supplementary Figure 5 Kaplan–Meier DMFS (a-c), RRFS (d-f) and OS (g-i) curves comparing IC+CCRT and CCRT alone for different risk groups in the training cohort

Abbreviations: CCRT, concurrent chemoradiotherapy; DMFS, distant metastasis-free survival; IC, induction chemotherapy; OS, overall survival; RRFS, regional relapse-free survival.

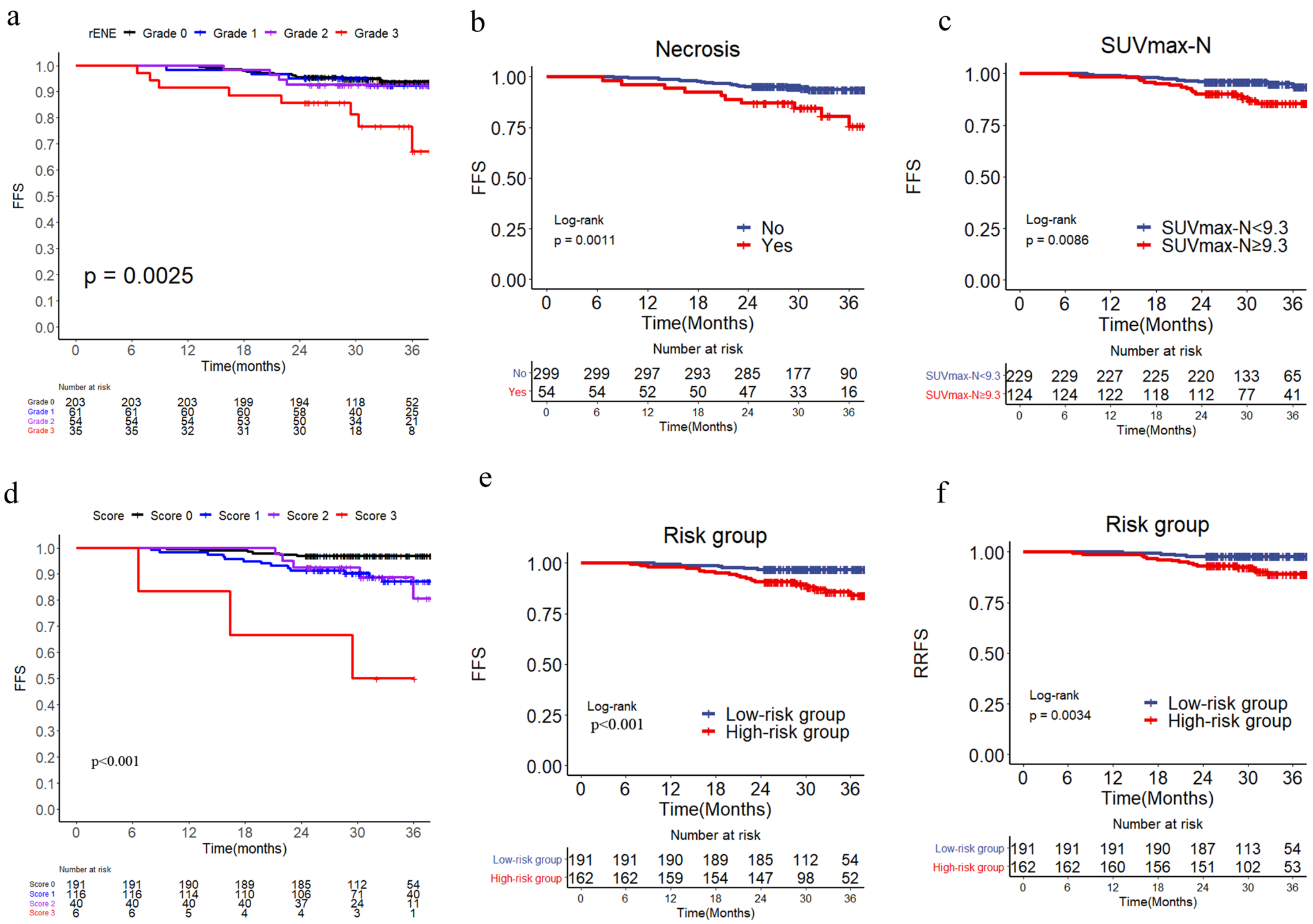

a
rENE Grade 0 Grade 1 Grade 2 Grade 3
FFS
p = 0.0025
Time(months)
Number at risk
Grade 0 203 203 203 199 194 118 52
Grade 1 61 61 60 60 58 40 25
Grade 2 54 54 54 53 50 34 21
Grade 3 35 35 32 31 30 18 8
b
Necrosis
FFS
Log-rank
p = 0.0011
No
Yes
Time(Months)
Number at risk
No 299 299 297 293 285 177 90
Yes 54 54 52 50 47 33 16
Time(Months)
c
SUVmax-N
FFS
Log-rank
p = 0.0086
SUVmax-N<9.3
SUVmax-N≥9.3
Time(Months)
Number at risk
SUVmax-N<9.3 229 229 227 225 220 133 65
SUVmax-N≥9.3 124 124 122 118 112 77 41
Time(Months)
d
Score Score 0 Score 1 Score 2 Score 3
FFS
p<0.001
Time(months)
Number at risk
Score 0 191 191 190 189 185 112 54
Score 1 116 116 114 110 106 71 40
Score 2 40 40 40 40 37 24 11
Score 3 6 6 5 4 4 3 1
e
Risk group
FFS
Log-rank
p<0.001
Low-risk group
High-risk group
Time(Months)
Number at risk
Low-risk group 191 191 190 189 185 112 54
High-risk group 162 162 159 154 147 98 52
Time(Months)
f
Risk group
RRFS
Log-rank
p = 0.0034
Low-risk group
High-risk group
Time(Months)
Number at risk
Low-risk group 191 191 191 190 187 113 54
High-risk group 162 162 160 156 151 102 53
Time(Months)

Supplementary Figure 6 Kaplan–Meier survival curves in the validation cohort

FFS curves of rENE (a), necrosis (b), SUVmax-N (c), risk scores (d), and risk groups (e) in the validation cohort; the RRFS curve of the different risk groups in the validation cohort (f). Abbreviations: FFS, failure-free survival; rENE, radiologic extranodal extension; RRFS, regional relapse-free survival; SUVmax-N, the maximal standardized uptake value of lymph node.
